# Delirium in your house: a survey during General Practitioner-programmed home visits

**DOI:** 10.1007/s40520-021-01806-1

**Published:** 2021-02-18

**Authors:** Lucio Tremolizzo, Lorena Bargossi, Benedetta Storti, Carlo Ferrarese, Giuseppe Bellelli, Ildebrando Appollonio

**Affiliations:** 1 School of Medicine and Surgery, University of Milano-Bicocca, building U8, room 2043 - via Cadore 48, 20900 Monza, MB Italy; 2 Neurology Unit, San Gerardo Hospital, Monza, Italy; 3 Acute Geriatric Unit, San Gerardo Hospital, Monza, Italy; 4 Milan Center for Neuroscience (Neuro-MI), Milan, Italy; 5 ATS Brianza, Monza, Italy

**Keywords:** Delirium, Home visits, General practitioner, 4AT, Dementia

## Abstract

**Objectives:**

To assess the prevalence of delirium (DEL) among older patients living at home and periodically visited by their General Practitioners (GPs).

**Design:**

Observational study.

**Setting:**

In Italy, programmed home visits by the GPs are regularly scheduled for their vulnerable and frail patients who are often on poly-drug regimens and suffering from dementia.

**Participants:**

*N* = 102 patients among those receiving programmed home visits by *n* = 6 GP based in the Brianza area (Lombardy).

**Measurements:**

Patients were screened for delirium with the Italian version of the 4AT, with a score ≥ 4 considered as a positive indicator for DEL. The Charlson Comorbidity Index (CCI), the Short Physical Performance Battery (SPPB), the presence of dementia, and benzodiazepine (BZD) use were recorded.

**Results:**

DEL+ was detected in almost half of the recruited sample (44.1%), and it was clearly associated with increased comorbidity and decreased motor abilities. Pre-existing dementia was documented in most of DEL+ patients (71.1%), while this was the case for only a minority of DEL- (5.2%, *p* < 0.00001). Analogously, BZD use was over-represented in the DEL+ group with respect to the DEL− one (73.3% *vs.* 22.8%, *p* < 0.00001).

**Conclusions:**

DEL prevalence as detected by GP during programmed home visits is surprisingly high, and related to motor impairment, comorbidities (among which dementia), and BZD use. DEL prompt recognition should be one of the goals of GP-programmed home visits, since this treatable and preventable condition is associated to an elevated burden of frailty and risk of death.

## Introduction

According to the Diagnostic and Statistical Manual of Mental Disorders, Fifth Edition (DSM-5) criteria [1], delirium is an acute disturbance in attention (i.e., reduced ability to direct, focus, sustain, and shift attention) and awareness (i.e., reduced orientation whether in the environment) that develops in a relatively short time-period (usually hours or days). Delirium is almost always triggered by a medical condition or inappropriate use of drugs and is associated with several negative outcomes and high costs of care [2–4].

Delirium is one of the most common acute mental disorders that affect the older people. In acute hospitals, delirium affects nearly one in five patients, but both prevalence and incidence is even higher in surgical wards and in intensive care unit, reaching a prevalence of 80% in mechanical ventilated patients [5]. Delirium is also common in the rehabilitation wards and in nursing homes [6, 7, 3].

To date, only a few studies have assessed the prevalence of delirium at home. The East Baltimore Survey [8] found an age-specific prevalence of 10.9 (95% CI 0.0–22.5) per 1,000 persons aged 55 years and older. However, it is not clear if any of these cases had co-existent dementia. In the Girona study [9], a door-to-door survey of 1,460 individuals aged 70 years and older, the delirium prevalence was 9.6 [95% CI 4.4–14.9] per 1,000 persons. In the Canadian Study of Health and Ageing (CSHA study [10] the point-prevalence of delirium was 6.3 (95% CI 4.1–9.6) per 1,000 persons. However, diagnoses of delirium and dementia were considered mutually exclusive, implying an underreport of the true delirium prevalence. In fact, pre-existing dementia is a well-known risk factor for delirium occurrence [11]. Recently, Manni and colleagues reported a retrospective cohort study of 2995 older patients referred to a Memory Clinic, finding that the rate of delirium was 3.64%, worsening functional and cognitive status at 6 months compared to baseline [12]. This apparent rarity of delirium in the extramural setting might be ascribed to the absence of hospital precipitating factors, such as being in an unfamiliar place or the change in the personal daily routine. Nevertheless, considering the high risk of missing delirium in a hospital ward setting [13], it is also possible that the true prevalence in the general population, or at least in specific subgroups of the elderly at home, might be higher than reported so far.

In Italy, General Practitioner (GP)-programmed home visits are regularly performed to check on a special subset of the general population characterized by elevated frailty, moderate to severe disability and chronic conditions that can be managed at home. During the programmed home visits, delirium can be easily missed, if not specifically focusing on it, and especially in the hypoactive form. This information, however, is conceivably of extreme value since delirium is a further important marker of frailty [14], rather than a transient and fully reversible condition associated only with acute insults, as already previously discussed by other Authors [9].

The aim of this observational study was to assess the prevalence of delirium among a cohort of older patients living at home and periodically visited by their GPs due to the elevated burden of their medical conditions.

## Patients and methods

Following ethical approval, *n* = 102 consecutive patients were recruited among those receiving programmed home visits by *n* = 6 GPs based in the Monza-Brianza area (mainly rural zones in the North-Western part of the Lombardy region in Italy). All patients were followed according to this modality because of either, significant logistic/motor difficulties in transporting them to the GP’s office [although truly bedridden patients were *n* = 11 (10.8%)], or because considered too fragile for moving. Most patients had more than one significant medical condition, but the main reason for being included in this program were: previous stroke (*n* = 19); severe cardiovascular disease (*n* = 8); COPD/severe respiratory problems (*n* = 8); advanced Parkinson’s disease (*n* = 7); dementia (*n* = 7); severe osteoarthrosis/orthopedic problems (*n* = 20); diabetic polyneuropathy (*n* = 14); age > 95 y.o. (*n* = 13); age > 85 y.o. and no relatives available (*n* = 6). These visits were programmed on a monthly basis.

All patients were screened for delirium by the GP with the Italian version of the 4AT, and a score ≥ 4 was considered as a positive indicator for delirium (DEL+) [15]. In case of a positive screening, the presence of delirium was further confirmed by clinical impression and opportune correcting maneuvers were taken by the GP on a case-by-case basis (e.g., programming blood or urine tests, changing setting details, prescribing drugs, etc*.*). The Charlson Comorbidity Index (CCI) [16] and the Short Physical Performance Battery (SPPB) [17] were also administered during the same visit. The presence of dementia known as one major predisposing factor of delirium, was recorded. Finally, benzodiazepine use (BZD, presence *vs.* absence) was recorded as well, since this delirium-predisposing factor has received in the more recent years, important attention in terms of public health and the de-prescription of these drugs is currently considered as a major goal of the GP activity [18].

Data are reported as mean ± standard deviation (range). Differences between two groups (DEL+  vs. DEL−) were calculated by the unpaired two-tailed Student’s *t* test while differences among more than two groups by the ANOVA followed by Tukey’s multiple comparisons test. Differences between categorical variables were assessed by the χ^2^ test. Correlations between variables were calculated with the two-tailed Pearson’s *r* test.

## Results

*N* = 33 male and N = 69 female patients were recruited. Mean age was 84.9 ± 7.9 (50–97) years-old and average education was 6.1 ± 2.0 (5–13) years [Verhage education score [19]: 2.6 ± 1.1 (2–6)]. Dementia was already present in *n* = 35 patients (34.3%) and BZD use was recorded in n = 46 patients (45.1%).

The 4AT scale could be administered to all patients [4.6 ± 4.0 (0–12)] and a score suggesting delirium (i.e., ≥ 4) was obtained in almost half of them [*n* = 45 (44.1%)]; all cases were subsequently confirmed by GP clinical impression. The CCI was 4.8 ± 2.3 (1–12) and the SPPB score was 3.1 ± 2.9 (0–12), accordingly to the expected decrease in the functional status of this selected population.

Demographic and clinical characteristics of patients with delirium with respect to those without are shown in Table 1 (DEL+ *vs.* DEL−). DEL+ patients did not differ from DEL− patients for the baseline demographic characteristics, but they displayed a significantly higher comorbidity score (on average + 3.9 CCI points, *p* < 0.0001) and decreased motor abilities (on average -2.3 points at the SPPB score, *p* < 0.0001). Accordingly, the 4AT score correlated with both the CCI (*r* = 0.43 *p* < 0.0001) and the SPPB one (*r* = 0.38 *p* < 0.0001).

**Table 1:** Clinical and demographic characteristics of patients with and without delirium

	DEL+ *n* = 45	DEL− *n* = 57	*p* value
4AT, score	8.5 ± 2.8 (4–12)	1.5 ± 1.0 (0–3)	*< 0.0001
Sex, M (%)	16 (35.5%)	17 (29.8%)	0.539
Age, y.o	83.5 ± 8.9 (50–97)	86.0 ± 6.9 (63–96)	0.106
Education, years	6.3 ± 2.4 (5–13)	5.9 ± 1.6 (5–13)	0.347
CCI, score	6.1 ± 2.3 (1–12)	3.8 ± 1.8 (1–11)	*< 0.0001
SPPB, score	1.8 ± 2.4 (0–8)	4.1 ± 2.8 (0–12)	*< 0.0001
Dementia, yes (%)	32 (71.1%)	3 (5.2%)	*< 0.00001 χ^2^ 48.3
BZD, yes (%)	33 (73.3%)	13 (22.8%)	*< 0.00001 χ^2^ 23.9

*BZD* use of benzodiazepine; *CCI* Charlson Comorbidity Index; *DEL*+  presence of delirium; *DEL*− absence of delirium; *SPPB* Short Physical Performance Battery

Pre-existing dementia was documented in most of DEL + patients (71.1%), while this was the case for only a minority of DEL- (5.2%, *p* < 0.00001). Dementia patients versus cognitively spared ones had a significantly higher 4AT score [8.6 ± 3.2 (0–12) vs. 2.4 ± 2.4 (0–12), *p* < 0.0001], increased comorbidity [CCI: 5.8 ± 2.2 (1–12) vs. 4.2 ± 2.2 (1–11), *p* = 0.0008], and decreased physical performances [SPPB score: 1.7 ± 2.4 (0–8) vs. 3.7 ± 2.8 (0–12), *p* = 0.0006]. Delirium was detected in *n* = 32 dementia patients out of 35 (91.4%) with respect to *n* = 13 out of 67 (19.4%) in cognitively spared ones (χ^2^ 48.3 *p* < 0.00001).

**Fig. 1:**
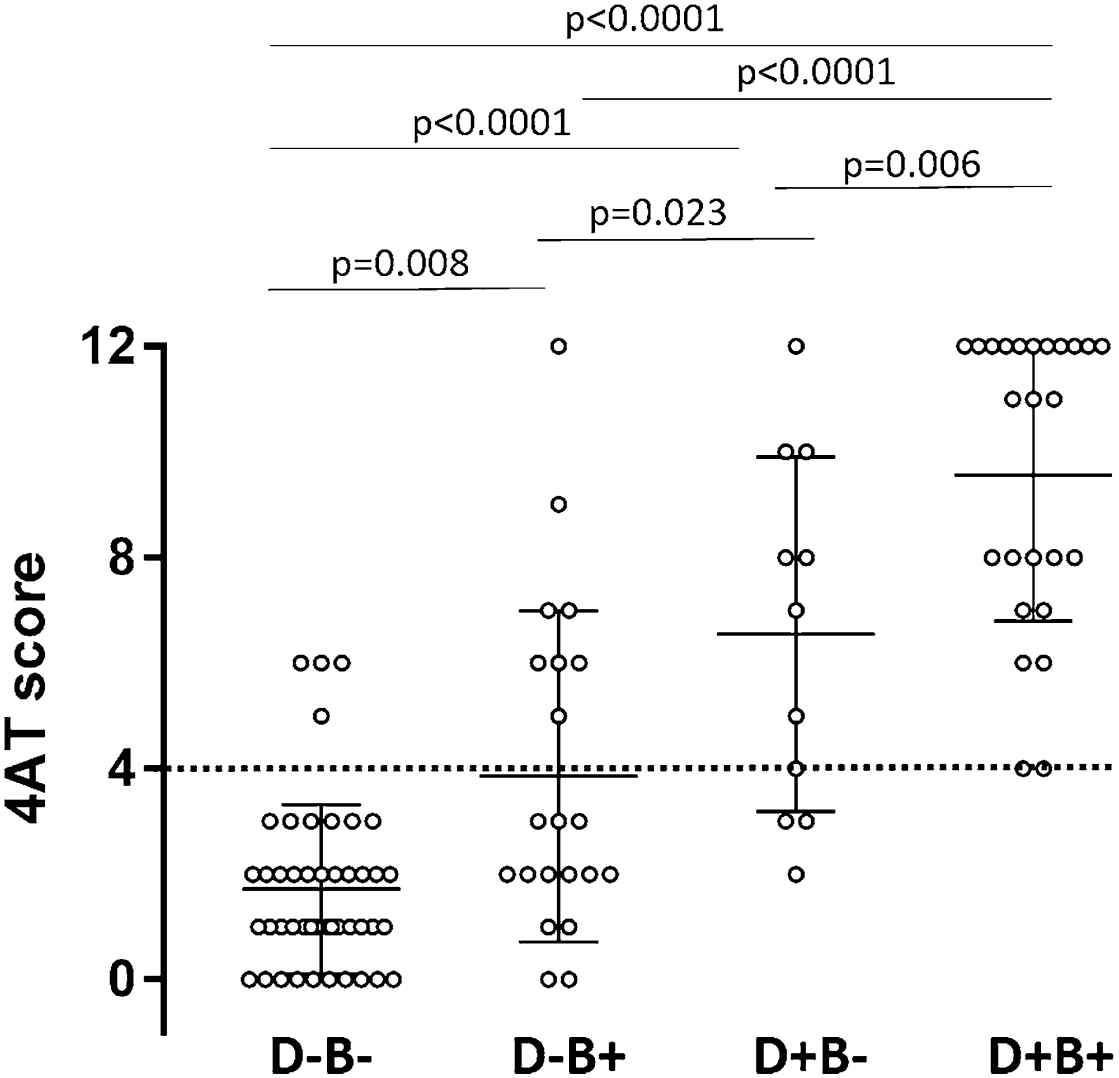
Delirium home-detected patients are mostly affected by dementia and BZD users. 4AT score according to the presence of both dementia (D+ vs. D−) and/or benzodiazepine use (B+ vs. B−). ANOVA *p* < 0.0001, followed by Tukey’s multiple comparison test. Test for linear trend: slope 2.6 *p* < 0.0001

Analogously, BZD use was over-represented in the DEL + group with respect to the DEL- one (73.3% vs. 22.8%, *p* < 0.00001). And again, BZD users versus non-users had increased 4AT score [6.9 ± 4.0 (0–12) vs. 2.6 ± 2.8 (0–12), *p* < 0.0001], increased comorbidity [CCI: 5.9 ± 2.4 (1–12) *vs.* 3.8 ± 1.7 (1–9), *p* < 0.0001], and decreased physical abilities [SPPB score: 3.5 ± 2.7 (0–8) vs. 3.5 ± 2.9 (0–12)]. Delirium was detected in *n* = 33 out of 46 BZD users (71.7%) with respect to *n* = 12 out of 56 (21.4%) non-users (χ^2^ 25.9 *p* < 0.00001). Finally, Fig. 1 shows a clear increase in the 4AT score stratified according to the presence of both BZD and dementia.

## Discussion

In this paper, we investigated the prevalence of delirium in home-based patients requiring periodic visits by their GPs due to the elevated burden of disease and their limited possibility of being managed by outpatient facilities. Conceivably, this peculiar subgroup of the general population is characterized by the most elevated degree of frailty among home-based patients and is therefore the natural target for improving the identification and management of critical situations. Delirium is highly prevalent among frail patients and represents by itself a critical condition that needs a prompt recognition to avoid major complications or death. Most studies addressed delirium prevalence and causes in the hospital setting, acute by definition, but the impact of a putative delirium prevention strategy in home-based patients may have greater magnitude. Therefore, since few data are available for home-based patients, and none specifically, for the subpopulation investigated by the present study, we sought first to address the real prevalence of the problem.

We found a surprisingly elevated prevalence of delirium in these patients (44.1%). Coherently, the 4AT score significantly correlated with both the CCI and the SPPB: as expected, patients with more comorbidities (and drugs) and more impaired physical performance are at higher risk for delirium. The presence of bedridden patients may have led to overestimate delirium prevalence but, plausibly, this is true only for a minority of patients (10.8%). One further limitation consists in the fact that delirium has been ascertained based only on one single 4AT assessment. However, in previous studies, the 4AT has shown good sensitivity and specificity toward DSM criteria, suggesting that is a reliable tool to detect delirium [20]. In addition, no formal power calculation was a priori performed, albeit the recruited population is quite consistent (*n* = 102). Thus, even when critically evaluating all these issues, the rate of delirium in our home-based patients receiving periodic GP visits appears unexpectedly high.

The exact relationship between dementia and delirium is a current matter of debate, but dementia is commonly considered the leading risk factor for delirium [21]. In our population, 71.1% of those patients experiencing delirium had a preexisting dementia and, even more revealing, almost all of cognitively impaired patients (91.4%) were found positive for delirium. Certainly, the distinction between delirium and dementia may be a hard task [22] and it becomes progressively harder with the worsening of cognitive decline. In any case, due to the fact that dementia and delirium are strictly interrelated (*i.e.*, the more severe is the degree of dementia the higher is the likelihood of developing delirium), GPs need to be formally trained in detecting delirium among their patients cared at home. In fact, delirium is a strong predictor of functional dependence, institutionalization, and mortality in older patients admitted to rehabilitation [23], and to the hospital [24]. In this perspective, the 4AT, due to its brevity and pragmatism may be particularly useful.

The literature indicates a weak relationship between BZD use and increased risk of delirium, in particular, outside of the ICU setting [25], and their use was specifically recorded in this study since family medicine guidelines now recommend the de-prescription of these drugs, mainly due to the elevated risk of addiction that they imply [18]. The results of our study reinforce these previous observations: among patients suffering from delirium, 73.3% were BZD user and, 71.7% of BZD users experienced delirium. In our sample, all BZD+ patients were long-term users (< 6 months). BZD use alone, however, moderately increased the risk of delirium when compared to dementia and the most striking effect was observed in dementia patients taking BZD, since all of them had a 4AT score suggestive for delirium. According to these observations, 4AT seems to be particularly informative when used to evaluate cognitively unimpaired patients assuming BDZ. Although BZD use is not recommended in the elderly population, and even more specifically in those subjects affected by dementia, these drugs continue to be prescribed all over the world, representing a problem of public health [25]. Our data support the idea that educational training is required to teach GPs that delirium may be precipitated or even worsened by BZD use in older patients [26]. Accordingly, both the American Geriatric Society, and the recent Scottish Intercollegiate Guidelines (https://www.sign.ac.uk/, March 2019) recommend BZD deprescription for reduction and management of delirium.

One major limitation of our study consists in the lack of information on sedative drugs other than BZD. In fact, neuroleptics could be potential confounders, and the global anticholinergic burden of the poly-drug regimens taken by our patients represent a definite source of risk for developing delirium, and, therefore, a potential unaddressed source for bias in our study [27].

As conclusion, our study focuses on delirium from the peculiar prospective of very frail patients in their own home, reporting for the first time a substantial prevalence of the phenomenon. This severe underestimation may have important consequences in terms of public health management since delirium is a harmful condition that can be prevented using multicomponent interventions [22] and can be rapidly screened for by GPs, using the 4AT tool. Furthermore, the recent SARS-CoV2 pandemic has strongly transformed medical practice; many hospitals have run out of beds, leading to the necessity of reducing hospitalization and developing home visit programmes. What is more, the very latest exponential rise of the pandemic may result in a further upward trend of delirium occurrence at home [28], leading to the need for developing effective programs for prevention, detection and treatment of this syndrome.
